# Coblation plus photodynamic therapy (PDT) for the treatment of juvenile onset laryngeal papillomatosis: case reports

**DOI:** 10.1186/1477-7819-12-275

**Published:** 2014-08-29

**Authors:** Chengyong Zhou, Baochun Sun, Feng Wang, Zhiyao Dai, Zeli Han, Jiahong Han, Maomao Chen, Yao Shen

**Affiliations:** Department of Otolaryngology, The First Affiliated Hospital of PLA General Hospital, No.51, HaiDian Distric Fu Cheng Road, Beijing, 100048 China

**Keywords:** juvenile onset laryngeal papillomatosis, JLP, coblation, photodynamic therapy, PDT, laryngeal papillomatosis

## Abstract

**Background:**

In treating juvenile-onset laryngeal papillomatosis, the most difficult aspect is preventing recurrence. After a single treatment, recurrence can begin after as soon as 20 days and the recurrent rate can be higher than 90%. The causes of recurrence include the presence of mucosal cells infected with papilloma virus, which are undetectable with the naked eyes, and surgery-induced infection. Photodynamic therapy (PDT) could effectively solve this problem. Virus-infected cells have a very high metabolic energy for capturing and internalizing the photosensitizer, which, after light stimulation, subsequently induces active oxygen species inside the nucleus, which kill infected cells. The second generation of photosensitizer agents (PA) are locally applied to avoid the intravenous systemic damage caused by first-generation PAs, and this method is widely used for the treatment of genital warts to very good effect.

**Methods:**

We used the photodynamic method to treat laryngeal papillomatosis in children and obtained significant efficacy. We followed three juvenile subjects with recurrent laryngeal papillomatosis through a course of treatment (each course includes three PDT sessions), with a follow-up after 6 months.

**Results:**

The characteristic procedures involve exposing the larynx with a laryngoscope and using low-temperature plasma technology to visualize the tumor resection, as the effects of plasma technology can reduce postoperative laryngeal edema and reduce intraoperative metastasis. PDT was performed during the first surgery, 20 days after and 30 days after surgery. At the 6-month follow-ups, there was no recurrence.

**Conclusion:**

This was the world's first successful reported case of the use of PDT treatment for juvenile laryngeal papillomatosis.

## Background

Laryngeal papillomatosis is the presence of non-invasive benign epithelial tumors, which includes both adult and juvenile-onset laryngeal papillomatosis (JLP) [1]. Multiple laryngeal papillomatosis is usually seen in children. The papillomas develop fast and are susceptible to relapse, but rarely in a malignant transformation [2, 3]. The overall prevalence of JLP is 3.6 to 4.3 per 100,000 children, 80% of whom are under 7 years old. JLP is mostly seen in children younger than 4 years old [4]. The onset of JLP is associated with human papillomatosis virus (HPV) [5]. JLP is transmitted from mother to fetus, i.e. disease onset is observed after months or years of incubation after the infants were delivered and infected by a mother with condyloma acuminate [6, 7]. Most young patients are not cooperative in laryngoscopy, which may lead to a delayed diagnosis. Most of the cases are found in the advanced stage and the scope of disease is extended. The major treatment for this disease is mainly tumor excision with a pedestal laryngoscope. Auxiliary treatments, such as CO_2_ laser, cryotherapy, electric cauterization, ultrasound therapy and chemotherapy, are also available [8–11].

The causes of recurrence include mucosal cells infected with the papilloma virus, which are undetectable with the naked eyes. Photodynamic therapy (PDT) could effectively solve this problem. PDT is a form of phototherapy that uses nontoxic light-sensitive compounds; when the nontoxic compounds are exposed selectively to light, they destroy targeted cells, such as microbial or cancer cells [12]. It is widely used clinically to treat different medical conditions such as infections and cancers [13, 14]. In 1998, Shikowitz [15, 16] attempted to use a photosensitizer intravenously to treat respiratory papillomatosis. But the intravenous application resulted in severe damage to the skin, so that the method cannot be widely used. With the application of second-generation photosensitizers, we can completely eliminate the systemic side effects of the damage. In recent years, the application of second-generation photosensitizers in clinical applications has brought revolutionary progress, decreasing the recurrence rate from 70% to 90%, to 10% [17]. The success is because PDT can remove the virus from mucosa without systemic side effects. As the mechanisms of virus infection and recurrence of laryngeal papillomatosis are almost the same among children, we intended in the present study to use PDT to treat laryngeal papillomatosis. Removal of laryngeal papillomas was initially accomplished with cold equipment to avoid thermal damage, thus preventing postoperative laryngeal edema or vocal cord hardening or adhesion. In three cases, PDT was performed during the first surgery, 20 days after and 30 days after surgery. To our surprise, at the 6-month follow-up, there were no cases of recurrence of JLP.

## Case presentation

### Anesthesia

For children with an existing tracheostomy, the intubation tracheal tube was directly replaced with an anesthetic tube. For children with category 3 dyspnea and difficulty breathing, a tracheotomy was performed under local anesthesia, followed by insertion of a cuffed tracheal tube and systemic anesthesia. This method could also prevent metastasis of laryngeal papillomatosis to the trachea during intubation. For children with category 2 dyspnea, oral intubation was used for anesthesia.

### Operating procedure

After general anesthesia, a self-retaining laryngoscope was targeted on the papillomatosis and the surrounding tissues. A low-temperature plasma ablation system, EVac 70 Xtra Plasma Wand (Sunnyvale, CA, USA) was used to remove the visible lesions. The head could be operated to both cut and coagulate at a low temperature of 65°F, thus avoiding igniting the oxygen during anesthesia. The normal mucous membrane was protected to a maximal extent. We used the endoscope at 0, 30 and 70 degrees to confirm if there were residual lesions after adequate hemostasis.

### Photodynamic therapy

The procedure was similar to that of our previous study [14]. The laser wavelength was 635 nm and the power was 280 to 300 mW. Aminolevulinic acid hydrochloride (topical powder, 3 to 5 bottles, 118 mg per bottle, Fudan-zhangjiang Bio-Pharmaceutical, Co, Ltd, Shanghai, China) was used for the PDT. Before application, the drug was diluted (0.5 ml for each 118 mg) to a final concentration of 20%. The solution was freshly prepared before each application and kept for not longer than 4 hr. After smearing the 20% aminolevulinic acid hydrochloride solution on the wound and surrounding mucosa, special attention was paid to the epiglottic vallecula, ventriculus laryngis, hypolarynx, sinus piriformis and other irregular cavity gaps. A cotton ball was immersed in the solution and placed on the surface of the papillomatosis, with a fresh solution re-applied to the cotton ball every 30 minutes. The medication was completed within 2 hr. The applied areas were kept out of direct light. Two hr after administration, a dual FD-400-A Laser (Wuhan Linyun Photoelectronic System Co, Ltd, Wuhan, China), with a wavelength of 635 nm and laser energy of 100 to 200 J/cm^2^, was placed 1 cm below the glottis so the lesion was fully covered by light. The irradiation lasted for 30 minutes.

### Photodynamic therapy interval and frequency

A return visit was made 1 to 2 weeks after the treatment, and PDT was carried out again if recession of the mucosa edema was observed. Coblation was applied if there was a residual lesion or recurrence. There were in total three sessions of PDT. If no recurrence was observed after 6 months, this was determined to be a cure. The tracheostomy tube was then removed.

### Case 1

**Figure 1:**
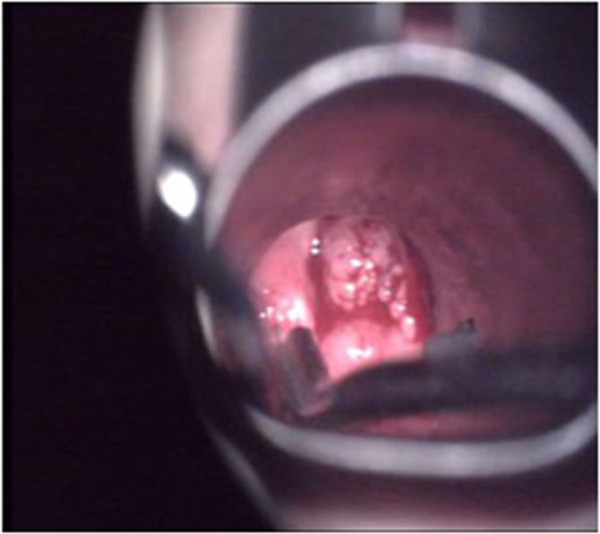
**Papillomas viewed by a self-retaining laryngoscope.**

**Figure 2:**
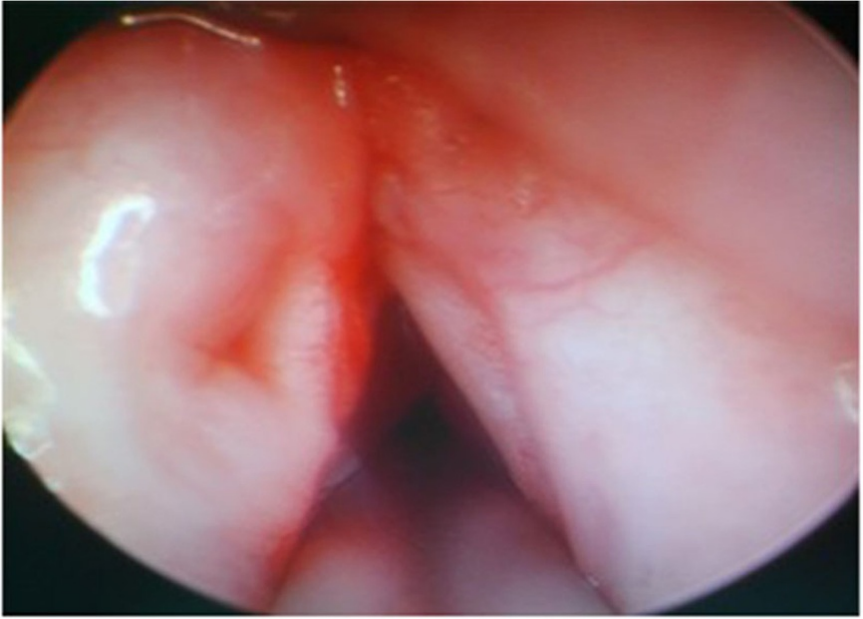
**Smooth vocal chords after three sessions of treatment.**

Case 1 was a 2-year-old girl, who was admitted due to repeated recurrence of JLP for 1 year after surgery. The girl received laser ablation for JLP under general anesthesia with a self-retaining laryngoscope seven times successively in a local hospital; recurrence was observed about every 20 days. A tracheotomy was performed due to extensive lesions that were found in the hypolarynx after the final surgery. The girl was then sent to our hospital. A physical examination found that her general conditions were poor, the trachea incision on the middle was noted, and the tracheal casing pipe was *in situ* and unblocked. Coblation for JLP with a self-retaining laryngoscope and PDT were given. Intraoperative findings showed papillomatosis on the bilateral ventriculus laryngis, right vocal cords and the areas from the hypolarynx to above the incision (Figure 1). Pathological findings after the operation were that the criteria for squamous epithelium cell papillomatosis were met, and the virus was subtype 11. PDT was given at once, and for the second and third times at 2 and 6 weeks after surgery. No recurrence of JLP was found during surgery (Figure 2). The follow-up was performed after 6 months, and no recurrence was found. The extubation was recently planned to be carried out.

### Case 2

Case 2 was a 2-year-old girl, who was admitted due to the repeated occurrence of papillary neoplasms on the pharynx oralis for 2 years. Her mother had condyloma acuminate. A physical examination showed a papillary neoplasm in the oral cavity above the bilateral tonsils, on the right wall of the pharynx and laryngeal surface, with a maximum size of 0.3 × 0.5 cm. Coblation for JLP with a self-retaining laryngoscope and PDT were given. The pathological findings after the operation were that the criteria for squamous epithelium cell papillomatosis were met, and the virus was subtype 16. The occurrence was found on the left tonsil 2 weeks after the first surgery, so a bilateral tonsillectomy and a second PDT were performed. PDT was performed for a third time and no occurrence was found 3 weeks after the surgery. The follow-up was performed after a year and there was no narrow pharyngeal cavity.

### Case 3

Case 3 was an 11-year-old boy, who was admitted due to post JLP surgery for over 20 days. Physical examinations in a local hospital found a papillary neoplasm on the bilateral vocal cords and ventriculus laryngis. The neoplasm was removed using a self-retaining laryngoscope. The pathological finding was papillomatosis with a low-grade intraepithelial neoplasia. Vocal cord adhesion was separated using a self-retaining laryngoscope and PDT was performed during, and 2 and 6 weeks after surgery. There was no papillomatosis recurrence at the 3-month follow-up.

### Discussion

Recurrent respiratory papillomatosis (RRP) is a chronic disease with a viral etiology that occurs in both children and adults. Many treatments have been tried, including both medical and surgical ones. However, there have been no cures. It was estimated that the incidence of RRP in the United States was 4.3 per 100,000 children [1] and 1.8 per 100,000 adults. In most pediatric cases, the time from onset of symptoms to diagnosis of RRP is approximately 1 year [18, 19], although the duration of symptoms may vary. Because it is extremely difficult to examine children, early symptoms such as hoarseness are often misdiagnosed as laryngitis. It is not until there is difficulty in breathing, is laryngoscopy used to make the correct diagnosis of laryngeal papillomatosis. Thus, once diagnosed, the surgery could be extremely difficult. The key to successful treatment is to remove all visible papillomas, without injuring normal tissues, to prevent recurrence.

At present, there is no cure for RRP. The current standard of care is surgical therapy with a goal of complete removal of papillomatosis and preservation of normal structures. In patients with anterior or posterior commissure disease or highly aggressive papillomatosis, the goal may be to clear the airway while preserving normal structures to avoid complications of subglottic and glottic stenosis, web formation, and resulting airway stenosis. Previously, this disease was treated by a combination therapy, such as cryotherapy, electric cauterization, ultrasound and chemotherapy. However, those treatments may cause severe damage to tissue, leading to tissue adhesion induced by secondary edema or a narrow respiratory tract. Laser ablation with a laryngoscope is a good alternative. After the papillomatosis has been removed, no damage to tissues or structures will be found. The carbon dioxide (CO_2_) laser has replaced cold instruments for removing RRP [20]. Even so, the recurrence rate after one operation is higher than 90%. The drawbacks of the CO_2_ laser are related to safety, as the laser beam may glance off nearby metal, such as a retractor, and injure the surgeon or patient. In addition, the laser smoke or plume has been found to contain active viral DNA, a potential source of infection [21]. Thus, smoke evacuators are necessary when this type of laser is used. The most serious safety concern with the CO_2_ laser is that the laser beam generates heat, which, if the beam inadvertently strikes the endotracheal tube in the oxygen-rich environment provided by anesthetic gases, could lead to an explosion or fire in the airway. Laser-generated heat could also cause injury to deeper tissues, leading to scarring with complications such as abnormal vocal cord function, spread of viral particles to previously unaffected areas and delayed local tissue damage.

Therefore, a good method to reduce thermal damage and prevent the spread of smoke with the flow of planting or with bleeding has been sought. Such a method would not lead to fire or instrument explosion. Over the past decade, low-temperature plasma surgery systems have been widely applied in clinical practice and have achieved good results [22]. Through an analysis of the cases above, it seems to us there are several advantages:

1. There might be mild mucosa edema when keeping the temperature of the surface tissue at 40 to 70°C during surgery.
2. There are minimal indirect tissue injuries and thermo-osmosis. The normal functions of the periphery tissue are protected when the lesions are removed, and mucosal injuries, postoperative papillomatosis growth and adhesion are reduced.
3. Hemostasis and ablation at the fixed points can be achieved via separation at the cellular level. Real-time rinsing and suction at the knife tip are used to reduce the transmission of HPV through blood.
4. Radiological hazards, extraneous odor and smoke are not produced during surgery. The potential hazards of aerosolization of the HPV virion for patients, medical staff and the operating room are prevented.

Therefore, the application of low-temperature plasma ablation for the treatment of JLP could not only improve the therapeutic effects, but also reduce the postoperative complications.

From 1998 to 2005, Shikowitz used the intravenous photosensitizer method to treat laryngeal and tracheal papillomatosis, and achieved encouraging efficacy. Although an efficacy-related intravenous photosensitizer dose was observed, due to the damage of the skin by the light, the dosage of the photosensitizer has been limited. Perhaps this is a constraining limitation on the full application of intravenous PDT photosensitizers. Since the second generation of photosensitizers, namely the local photosensitizer aminolevulinic acid hydrochloride, has been clinically used in recent years, the application of PDT for the treatment of condyloma acuminate (when infected by HPV) has achieved significant effects. This encouraged us to use PDT to remove JLP lesions. The rationale of PDT [23] is that when the photosensitizers in the human tissues are exposed to illumination at the correct wavelength, they absorb photon energy and transform into the excited state from the ground state. Photosensitizers in the excited state release energy rapidly through chemical deexcitation, during which a large amount of reactive oxygen is released, which interacts with various biomacromolecules and may destroy the HPV structure and functions, to achieve the therapeutic effects.

After the results of above cases, we consider that there are several advantages of PDT for the treatment of JLP:

1. The drugs used in PDT may penetrate into various irregular cavities of the cavum laryngis, and act on the whole mucosal tissue. The unique surface elimination mechanism may improve surgical treatment significantly compared to the point elimination of physical therapy such as lasers, cryotherapy, electric cauterization, etc.
2. This is an auxiliary surgical treatment, which could eliminate any potential HPV virus that could not be identified by the naked eyes and reduce the postoperative recurrence rate.
3. It selectively kills infected cells but does not harm normal ones. This significantly reduces laryngostenosis, hoarseness of the voice and other surgical complications.
4. Repeated application could significantly increase the cure rate and reduce the postoperative recurrence rate.

To completely eliminate the visible papillomatosis and its tissues, a tumorectomy was performed with a laryngoscope for all three child patients. We used the endoscope at 0, 30 and 70 degrees to observe the scope of the lesions, especially lesions of the hypolarynx for case 1 and lesions of the anterior commissure and its surrounding tissue for case 2. After treatment, we found the following advantages of treatment with a laryngoscope:

1. It is not necessary to observe the glottis because the self-retaining laryngoscope lifts the epiglottis up. Lifting the root of the tongue and the laryngeal examination can be performed at different angles and distances. The incidence of epiglottis tissue edema might be reduced due to the avoidance of stimulation and stress caused by the self-retaining laryngoscope on the epiglottis.
2. Through the adjustment of the angles, the laryngoscope can reach parts where a microscope cannot reach, such as the laryngeal surface, lower margin of the vocal cords and areas under the hypolarynx. Thus the papillomatosis could be accurately removed and there will be no collateral injuries.
3. The tracheal cavity examination and intratracheal tumorectomy are superior. The condition of the trachea may not be observed well by a microscope due to trachea cannula, while the laryngoscope can travel through the rima glottidis and hypolarynx and reach the trachea because of its smaller diameter. This allows for a more accurate examination and better lesion removal. Meanwhile, damage to the normal tissues might be minimized to some extent.

The operation time and the treatment course for JLP are very long. All three cases above had surgery under general anesthesia, and phototherapy could only be performed after 3 hr of medication; three sessions of treatment were required for each of them. Good coordination with the operation room is required due to the long occupation by the anesthetist. The medical expense of the whole course is high. JLP is transmitted from mother to fetus and is associated with HPV. This affects the privacy of the family and family members, and causes both mental and economic burdens. Therefore, the rate of lost to follow-up is high, especially for high-risk child patients. In addition, the repeated treatments and the mentality of the physician and parents may influence the physical and mental development of the child. The intervention and guidance from the medical, social, family and physiological points of view that are provided to the parents of patients in a critical condition will help them to maintain their faith and not give up. So, the great contribution of PDT is that it significantly shortens the treatment course for JLP and increases the cure rate.

It is worth mentioning that the amount of drugs administered was very small because of the local administration. We did not observe any photosensitive damage in other tissues. The drug was metabolized within 24 hr. Overall, the results of the treatments are very encouraging, and a literature review revealed that we were pioneers in the treatment of JLP with PDT.

## Conclusion

PDT treatment for juvenile laryngeal papillomatosis through local applied photosensitizer agent can obviously decrease the recurrence rate.

## Consent

Consent for article type Case report.

## Abbreviations

HPV: human papillomatosis virus
JLP: juvenile-onset laryngeal papillomatosis
PDT: photodynamic therapy
RRP: recurrent respiratory papillomatosis.
